# Decreased Nucleus Accumbens Connectivity at Rest in Medication-Free Patients with Obsessive-Compulsive Disorder

**DOI:** 10.1155/2021/9966378

**Published:** 2021-06-01

**Authors:** Yunhui Chen, Yangpan Ou, Dan Lv, Jidong Ma, Chuang Zhan, Ru Yang, Cuicui Jia, Tinghuizi Shang, Lei Sun, Yuhua Wang, Zhenghai Sun, Guangfeng Zhang, Xiaoping Wang, Wenbin Guo, Ping Li

**Affiliations:** ^1^Department of Psychiatry, Qiqihar Medical University, Qiqihar, Heilongjiang 161006, China; ^2^National Clinical Research Center for Mental Disorders, Department of Psychiatry, The Second Xiangya Hospital of Central South University, Changsha, Hunan 410011, China; ^3^Department of Psychiatry, Baiyupao Psychiatric Hospital of Harbin, Harbin, Heilongjiang 150050, China; ^4^Department of Radiology, The Second Xiangya Hospital of Central South University, Changsha, Hunan 410011, China; ^5^Department of Radiology, The Third Affiliated Hospital of Qiqihar Medical University, Qiqihar, Heilongjiang 161000, China

## Abstract

**Background:**

Patients with obsessive-compulsive disorder (OCD) experience deficiencies in reward processing. The investigation of the reward circuit and its essential connectivity may further clarify the pathogenesis of OCD.

**Methods:**

The current research was designed to analyze the nucleus accumbens (NAc) functional connectivity at rest in medicine-free patients with OCD. Forty medication-free patients and 38 gender-, education-, and age-matched healthy controls (HCs) were recruited for resting-state functional magnetic resonance imaging. Seed-based functional connectivity (FC) was used to analyze the data. LIBSVM (library for support vector machines) was designed to identify whether altered FC could be applied to differentiate OCD.

**Results:**

Patients with OCD showed remarkably decreased FC values between the left NAc and the bilateral orbitofrontal cortex (OFC) and bilateral medial prefrontal cortex (MPFC) and between the right NAc and the left OFC at rest in the reward circuit. Moreover, decreased left NAc-bilateral MPFC connectivity can be deemed as a potential biomarker to differentiate OCD from HCs with a sensitivity of 80.00% and a specificity of 76.32%.

**Conclusion:**

The current results emphasize the importance of the reward circuit in the pathogenesis of OCD.

## 1. Introduction

Obsessive-compulsive disorder (OCD) is a heritable, disabling, and chronic psychiatric disorder with an approximated lifetime prevalence of 1%–3% [1]. Patients with OCD may exhibit repetitive and uncontrolled behaviors (compulsions) to reduce the level of anxiety caused by recurrent and intrusive thoughts (obsessions) [2]. Hence, compulsions could be considered as addictive behaviors that are involved with deficient processing of rewards [2, 3].

The reward circuit is composed of the nucleus accumbens (NAc), anterior cingulate cortex (ACC), ventral tegmental area (VTA), hippocampus, and brain areas of the prefrontal cortex (PFC), which are involved in incentive salience, positive emotions, and associative learning [4, 5]. As a crucial brain reward region, NAc (part of the ventral striatum) mergers different excitatory and inhibitory inputs to the salience signals of rewarding stimuli and is a successful target for deep brain stimulation for OCD [6–8]. Patients with OCD display decreased reward anticipation activity in the NAc [2] and abnormal ventral striatal circuitry during reward-based spatial learning [9]. The NAc-orbitofrontal circuitry plays an important role in reward processing [10] and shows abnormal activity during reward processing and resting state in OCD [3, 11, 12]. Moreover, the functional connectivity (FC) strength between NAc and orbitofrontal cortex (OFC) can predict clinical symptoms of OCD [12]. Hence, patients with OCD may experience deficiencies in reward processing. Therefore, the investigation of the reward circuit and its essential connectivity may further clarify the pathogenesis of OCD.

In the current research, bilateral NAc was used as the seeds to conduct the seed-based FC in the reward circuit at rest in medicine-free patients with OCD. Based on previous researches, we expected that altered NAc FC would be found in some regions of the reward circuit at rest in patients with OCD. We also hypothesized that changed FCs might be correlated with the clinical characteristics of OCD and these characteristics could be used as potential biomarkers to distinguish patients with OCD from healthy controls (HCs).

## 2. Materials and Methods

### 2.1. Participants

This study was confirmed by the Qiqihar Medical University Research Ethics Committee. The study procedures were informed, and a written informed consent was signed by all participants.

Forty participants (27 males and 13 females) with OCD were enrolled from the Qiqihar Mental Health Center and Fourth Affiliated Hospital of Qiqihar Medical University, China. The patient version of the Structured Clinical Interview for DMS-IV (SCID) was used for diagnosis. The Yale–Brown Obsessive Compulsive Scale (Y-BOCS), Hamilton Anxiety Rating Scale (HAMA), and 17-item Hamilton Depression Rating Scale (HAMD) were used to evaluate the clinical symptoms of OCD. Twenty-two patients with OCD had a history of antipsychotic treatment, antiobsessive medication, or antidepressant use, whereas 18 individuals were drug naive. None of the patients took any psychotropic drugs for at least 4 weeks before brain image acquisition. Thirty-eight HCs (25 males and 13 females) matched in terms of gender, education, and age were recruited with the nonpatient version of SCID from the local community. All individuals were right handed and Han Chinese and had the same inclusion criteria as follows: (1) 18–50 years of age, (2) no serious physical disease and neurological or psychiatric illness, (3) no drug or alcohol dependence, (4) not pregnant, (5) no contraindication for a magnetic resonance imaging (MRI) scan, and (6) no movement distance of more than 2 mm or rotation angle of more than 2°. HCs were excluded if they had any first-degree relatives with mental diseases.

### 2.2. MRI Data Acquisition and Data Processing

For all individuals, resting-state functional MRI (rs-fMRI) was conducted using a 3.0-Tesla GE 750 Signa HDX Scanner (General Electric Healthcare, Waukesha, Wisconsin) at the Third Affiliated Hospital of Qiqihar Medical University. All participants were instructed to use foam pads and earplugs to reduce the scanner noise effect, remain in the supine position, close their eyes, relax, stay awake, and remain motionless (especially the head). The rs-fMRI data were procured via an echo-planar imaging (EPI) sequence with the following setup: repetition time = 2000 ms, axial slices = 33, echo time = 30 ms, slice thickness = 3.5 mm, interslice gap = 0.6 mm, flip angle = 90°, field of view = 200 × 200 mm^2^, data matrix = 64 × 64, and 240 volumes (8 min) in total. None of the participants displayed clinically significant structural abnormalities.

Data were preprocessed using the Data Processing and Analysis for Brain Imaging software [13]. The first 10 functional volumes were discarded to ensure a steady initial signal and adapt to the environment. Slice timing correction and head motion correction were performed for the 230 remaining EPI images. Realigned images were spatially normalized to a standard Montreal Neurological Institute space and resampled to 3 × 3 × 3 mm^3^. Afterwards, the realigned images were smoothed with a 4 mm full-width half-maximum isotropic Gaussian kernel. Subsequently, temporal bandpass filtering (0.01–0.08 Hz) was managed to eliminate the covariate effect of low-frequency drift and high-frequency physiological noise. The 24 motion parameters and signals from the cerebrospinal fluid and white matter were used as nuisance covariates. The identification of “band” time points was scrubbed using a threshold of 0.2 mm of framewise displacement (FD) and one back and two subsequent neighbors [14], and the mean FD for each subject was calculated.

### 2.3. Seed-Based Functional Connectivity Analysis

We selected bilateral NAc (from the Harvard Oxford Atlas) as regions-of-interest (ROIs) for the whole-brain FC analysis. This process was managed with the REST software [15]. For each subject, we obtained the averaging time series of all voxels in each ROI and Pearson's correlation analysis was carried out between the reference time courses of the ROI and the other voxels in the whole brain. The correlation coefficients were transformed to standard *z* values for normality. Then, the seed-based FC maps were established.

### 2.4. Statistical Analysis

Two-sample *t*-test was used to compare the demographics (such as age and years of education), clinical data (such as clinical scales), and FD. *Χ*^2^ test was conducted to compare the sex distributions of the two groups. The above processes were performed using SPSS version 23.0 (SPSS Inc., Chicago, IL, USA).

In the current study, we used two-sample *t*-tests based on a general linear model (GLM) to detect an effect that arises from one group versus another. The principle of the two-sample *t*-test based on GLM is to use multiple variables to predict a dependent variable through establishing a regression model (each variable has a corresponding regression coefficient) [16]. The two sample *t*-test was used to test whether the variable is statistically significant. The variable plays a small role in the regression model and is not a predictor of the dependent variable if the *p* value is greater than 0.05. On the contrary, the variable is a predictor of the dependent variable in the regression model if the *p* value is less than 0.05 [16]. Therefore, two-sample *t*-tests were used to compare group differences of the seed-based FCs in a voxel-wise way with the mean FD values, age, and sex as covariates of no interest. Gaussian random field theory corrected *p* < 0.05 was set as the significance level for multiple comparisons.

Pearson correlation was performed to investigate the relationship between altered FC values and clinical characteristics in OCD. The significance level of *p* < 0.05 (Bonferroni corrected) was used for the correlation results.

LIBSVM (library for support vector machines) [17] was conducted to examine whether altered seed-based FCs could distinguish OCD from HCs.

## 3. Results

### 3.1. Demographic and Clinical Data

The demographic and clinical data of the participants are displayed in Table 1. A total of 40 medication-free OCD and 38 HCs consented to enroll in this research. No difference was observed in terms of age (*t* = 0.05, *p* = 0.71), gender (*X*^2^ = 0.32, *p* = 1.00), educational level (*t* = 0.50, *p* = 0.83), and FD (*t* = 1.25, *p* = 0.13) between the two groups. Among the clinical characteristics, significant group differences were discovered in Y-BOCS (*t* = 25.27, *p* < 0.01), HAMD (*t* = 9.04, *p* < 0.01), and HAMA (*t* = 9.00, *p* < 0.01).

### 3.2. Group Differences of Seed-Based FC

**Figure 1:**
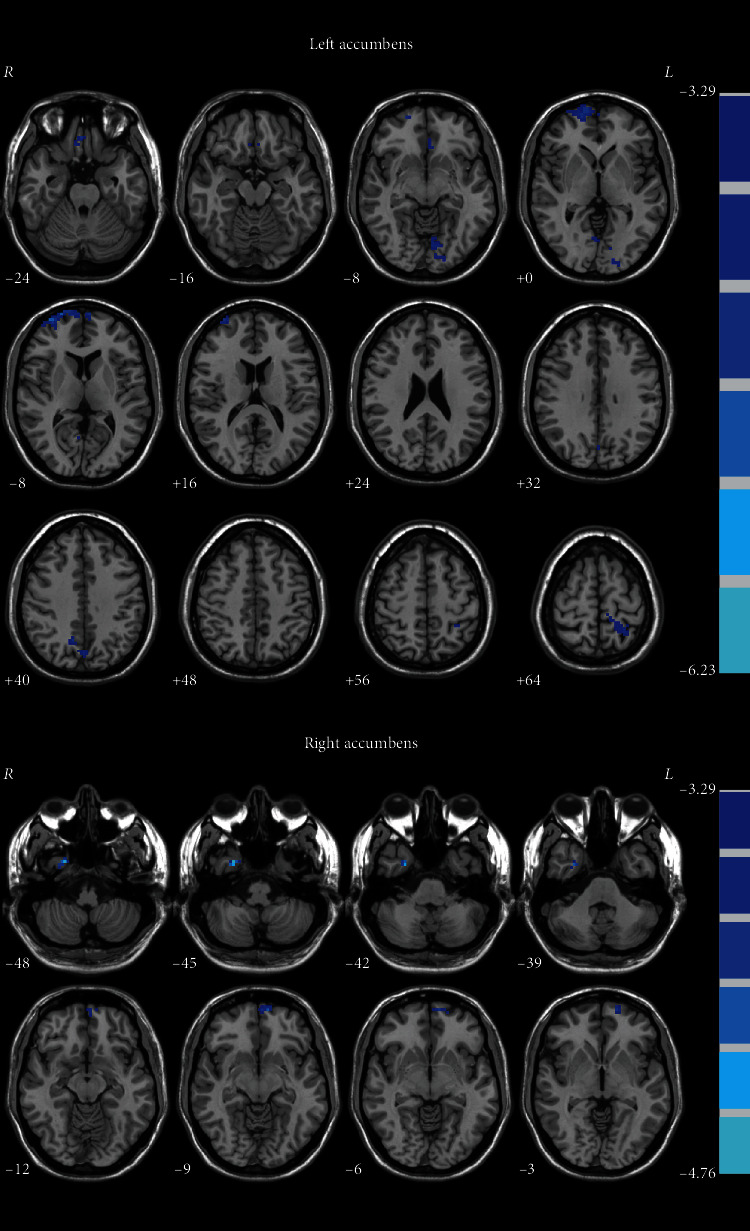
Brain regions with significant differences on seed-based functional connectivity in OCD. The color bar indicates the *T* values from two-sample *t*-tests. The blue color denotes decreased functional connectivity values in the patients. OCD: obsessive-compulsive disorder.

For the left NAc, patients with OCD displayed significantly decreased FC in the bilateral OFC, bilateral medial prefrontal cortex (MPFC), bilateral lingual gyrus, bilateral precuneus, and left superior parietal lobule (Table 2 and Figure 1). For the right NAc, OCD displayed significantly decreased FC in the left OFC and right fusiform (Table 2 and Figure 1).

### 3.3. Correlations between Altered Seed-Based FC and Clinical Characteristics in OCD

No correlations were found between the decreased bilateral NAc FC and clinical characteristics (e.g., Y-BOCS, HAMD, or HAMA subscale scores, age, illness duration, and education level) in patients with OCD.

### 3.4. LIBSVM Analysis

**Figure 2:**
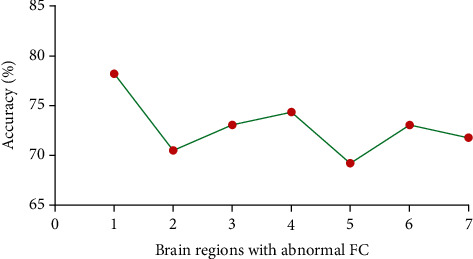
Accuracy (%) of abnormal FC values in a single brain region to discriminate OCD from HCs. FC: functional connectivity; OCD: obsessive-compulsive disorder; HCs: healthy controls; 1: bilateral medial prefrontal cortex; 2: bilateral lingual gyrus; 3: left superior parietal lobule; 4: bilateral precuneus; 5: bilateral orbitofrontal cortex; 6: left orbitofrontal cortex; 7: right fusiform.

**Figure 3:**
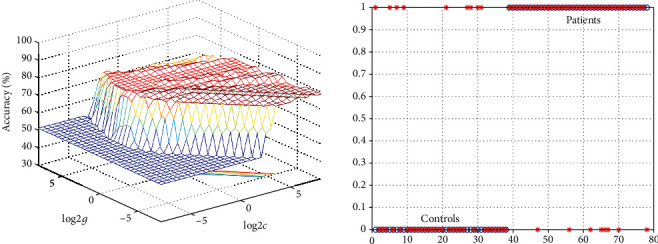
Visualization of SVM results by using FC values between the left NAc and the bilateral medial prefrontal cortex to differentiate patients with OCD from HCs. Left: 3D visualization of SVM with the best parameters; right: classification map of the FC values of the bilateral middle cingulate cortex; SVM: support vector machine; FC: functional connectivity; NAc: nucleus accumbens; OCD: obsessive-compulsive disorder; HCs: healthy controls.

As shown in Figures 2 and 3, seven brain regions with altered FCs (1 = bilateral MPFC; 2 = bilateral lingual gyrus; 3 = left superior parietal lobule; 4 = bilateral precuneus; 5 = bilateral OFC; 6 = left OFC; and 7 = right fusiform) were discovered in OCD. SVM analysis was computed using these seven brain regions. The classification accuracies are as follows: 1 = 78.21% (61/78), 2 = 70.51% (55/78), 3 = 73.08% (57/78), 4 = 74.36% (58/78), 5 = 69.23% (54/78), 6 = 73.08% (57/78), and 7 = 71.80% (56/78). The results of the SVM analysis showed that the decreased left NAc-bilateral MPFC connectivity could be used to classify OCD with a sensitivity of 80.00%, a specificity of 76.32%, and an accuracy of 78.21%.

## 4. Discussion

In the current research, we selected bilateral NAc as ROIs to examine the seed-based FCs at rest in medication-free patients with OCD. Consistent with our hypothesis, the primary result indicated significantly decreased FC values between the left NAc and bilateral OFC and bilateral MPFC and between the right NAc and left OFC in the reward circuit at rest in patients with OCD. Furthermore, decreased left NAc-bilateral MPFC connectivity can be used as a potential biomarker to differentiate OCD from HCs with optimum specificity and sensitivity. In addition, we found decreased FC between the bilateral NAc and parietal and occipital lobes, including the bilateral precuneus, left superior parietal lobule, bilateral lingual gyrus, and right fusiform. However, in contrast to our hypothesis, no correlations were observed between altered FC values and clinical characteristics in patients with OCD.

The NAc is an integral and complex hub in the reward circuit [7], and it connects the OFC and MPFC that constitute the cortico-striato-thalamocortical (CSTC) circuit in OCD [18, 19]. The deficiency in reward processing related to NAc abnormalities is involved in OCD [20]. In the current study, decreased NAc-OFC connectivity (such as left NAc-bilateral OFC and right NAc-left OFC) has been discovered. The OFC has a key role in expected reward valuation and behavioral planning [21], whereas the NAc is involved in modulating the behavior by focusing on the rewarding environmental stimuli [22]. Decreased FC between NAc and OFC may be correlated to the impaired coordination in transferring and evaluating the rewarding environmental stimuli and dysfunctions on planning and modulating behavior that may lead to driving obsessions and repetitive compulsions of OCD [21]. Decreased NAc-OFC connectivity suggests dysfunction in reward processing at rest in OCD [10]. However, a few studies discovered increased FC values between NAc and OFC at rest in OCD [3, 12]. The discrepancies between previous studies and the current results may be caused by the sample size and heterogeneity of the patients, such as illness duration, medication, symptom severity, and dimension [3, 23].

The MPFC generates cognitive and emotional information and plays a key role in emotional assessment and expression (especially negative emotion) within the reward circuit [24, 25]. The decreased FC between NAc and MPFC may underlie the dysfunctional correlation in the reward circuit at rest in OCD [4, 5, 26]. NAc may fail to merge the negative emotional signals with salience signals to modulate behavior, while MPFC may be unable to generate/assess cognitive and emotional information based on the rewarding environmental stimuli collection through the NAc. Therefore, the decreased FC between NAc and MPFC may be involved in the failure of the regulation and representation of negative emotion generated from obsessions and may contribute to the pathogenesis of negative emotions, such as anxiety and depression in patients with OCD.

SVM analysis manifests that the decreased left NAc-bilateral MPFC connectivity can be used to differentiate OCD from HCs with a specificity of 76.32%, a sensitivity of 80.00%, and an accuracy of 78.21%. As a multivariate method, the SVM method maximizes the boundary between classes in a high-dimensional space and considers each voxel as a spatially independent unit and can further be used to interpret the results of high discriminative power [27, 28]. In the medical field, a specificity and sensitivity value of 70% indicate a highly credible result [29]. Consequently, decreased left NAc-bilateral MPFC connectivity at rest can be deemed as a potential biomarker to distinguish OCD from HCs. In line with the current result, a previous research also manifested that the FC map of the MPFC can contribute to differentiate OCD [30].

In addition to the reduced FC values found in the reward circuit, we also discovered decreased FC values between bilateral NAc and parietal and occipital lobes. Numerous neuroimaging studies reported parietal lobe abnormalities in patients with OCD [31, 32]. The parietal lobe has a significant role in response inhibition and attentional set shifting that are damaged in OCD [33, 34]. The decreased FC value in the parietal lobe may be related to the impairment in attentional set shifting and response inhibition, which may contribute to the repetitive and uncontrolled obsession and compulsion of OCD. As a posterior brain region, the occipital lobe is involved in the pathogenesis of OCD [35–38]. The occipital lobe is associated with cognitive flexibility and fear/defensive and is involved in cognitive and emotional abnormalities in OCD [35]. Decreased FC values were observed in the occipital lobe at rest in OCD, suggesting that altered FC values in the large-scale brain system are not limited to the CSTC circuits at rest but may participate in the pathogenesis of OCD [36].

In a previous research, the FC between NAc and OFC is correlated with the clinical symptoms of OCD [12]. However, in contrast to our hypothesis, no correlations were found between altered FC values and the clinical parameters in OCD in the current research. We infer that the decreased NAc connectivity may be a trait alteration for OCD independent of the clinical characteristics [39]; moreover, the relatively small sample size of the current study might have caused the absence of correlation [40]. Furthermore, the Bonferroni correction may limit the correlations between altered NAc connectivity and clinical characteristics in the patients.

Several limitations in our study must be mentioned. First, the sample size was relatively small and the OCD samples were not separated into different clinical subtypes. Second, psychotropic medication (22 patients in our study had a history of psychotropic medication) may influence NAc connectivity at rest in patients with OCD. Third, the NAc connectivity during the task state associated with reward processing was not assessed in our study. Future research should combine the resting state and task state to explore the reward circuit and its essential connectivity in patients with OCD. Forth, the current research is a cross-sectional research. A longitudinal research warrants the investigation of the changes of the NAc connectivity after intervention. Finally, it is uncertain whether HCs did not have any “reward” system deactivated because these HCs could have had previous experience with pleasure seeking behaviors. However, monetary compensation was not provided to patients with OCD either, and thus, the two groups were matched regarding the resting state without monetary compensation.

Despite the limitations, our findings showed decreased NAc connectivity in the reward circuit at rest in medication-free patients with OCD. Decreased left NAc-bilateral MPFC connectivity may act as a potential biomarker for distinguishing OCD. The current results emphasize the importance of the reward circuit in the pathogenesis of OCD.

## Figures and Tables

**Table 1:** Demographic and clinical characteristics of participants.

	OCD patients (*n* = 40)	HCs (*n* = 38)	*X* ^2^/*t*	*p*
Age (years)	27.28 ± 8.16	27.18 ± 8.33	0.05	0.71
Sex (male/female)	27/13	25/13	0.03	0.87
Education (years)	13.40 ± 2.87	13.74 ± 3.03	−0.50	0.83
Illness duration (months)	66.68 ± 75.54			
Y-BOCS total score	24.90 ± 5.73	1.13 ± 0.88	25.27	<0.01
Y-BOCS obsessive thinking	12.85 ± 4.25	0.37 ± 0.49	17.98	<0.01
Y-BOCS compulsive behavior	12.05 ± 4.62	0.74 ± 0.72	14.92	<0.01
HAMD	8.05 ± 4.40	1.45 ± 0.95	9.04	<0.01
HAMA	10.83 ± 6.55	1.16 ± 1.00	9.00	<0.01
FD	0.04 ± 0.02	0.03 ± 0.01	1.25	0.13
Time points scrubbed out	1.13 ± 2.256	1.00 ± 2.418	0.25	0.95

OCD: obsessive-compulsive disorder; Y-BOCS: Yale-Brown Obsessive-Compulsive Scale; HAMD: 17-item Hamilton Depression Rating Scale; HAMA: Hamilton Anxiety Rating Scale; FD: framewise displacement. Variables of age, education, Y-BOCS total score, subscales score, HAMD score, HAMA score, and FD were tested by two-sample *t*-test; the results were indicated by *t* values. Categorical data such as gender was tested using a chi-squared test; the result was indicated by *X*^2^.

**Table 2:** Regions with abnormal functional connectivity with the accumbens in patients with OCD.

Cluster location	Peak (MNI)			Number of voxels	*T* value
	*x*	*y*	*z*		
*Seed: left accumbens*					
Bilateral OFC	3	39	−27	49	−4.4404
Bilateral MPFC	24	69	6	198	−6.2321
Bilateral lingual gyrus	−21	−96	−3	97	−4.5262
Bilateral precuneus	9	−72	42	62	−4.5731
Left superior parietal lobule	−27	−51	63	42	−3.9449
*Seed: right accumbens*					
Left OFC	−9	63	−9	33	−4.0701
Right fusiform	24	3	−48	37	−4.7571

The significance level was set at *p* < 0.05 for multiple comparisons corrected by Gaussian random field (GRF) theory (voxel significance: *p* < 0.001, cluster significance: *p* < 0.05). Age, sex, and the mean FD values were used as covariates to minimize the potential effects of these variables. MNI: Montreal Neurological Institute; OFC: orbitofrontal cortex; MPFC: medial prefrontal cortex.

## Data Availability

Our data may be available upon reasonable request. Please contact lipingchxyy@163.com for details.
